# Effects of Two-Way Cold Rolling and Subsequent Annealing on the Microstructure and Tensile Properties of Low-Carbon Steel with Different Initial Microstructures

**DOI:** 10.3390/ma19030466

**Published:** 2026-01-24

**Authors:** Toshio Ogawa, Hidetomo Hayashi, Hiroyuki Dannoshita

**Affiliations:** 1 Department of Mechanical Engineering, Faculty of Engineering, Aichi Institute of Technology, 1247 Yachigusa, Yakusa-cho, Toyota 470-0392, Japan; 2 School of Materials and Chemical Technology, Department of Materials Science and Engineering, Institute of Science Tokyo, S8-5, 2-12-1 Ookayama, Meguro-ku, Tokyo 152-8550, Japan

**Keywords:** two-way cold rolling, initial microstructure, recrystallization, strength–ductility balance, low-carbon steel

## Abstract

We investigated the effects of two-way cold rolling and subsequent annealing on the microstructure and tensile properties of low-carbon steel with different initial microstructures. Two types of hot-rolled sheet specimens were prepared: specimen P, consisting of ferrite and pearlite, and specimen M, consisting of martensite. The hot-rolled sheets were cold-rolled in two directions and subsequently annealed. Two-way cold rolling promoted shear-band formation compared with one-way cold rolling. Furthermore, the two-way cold-rolled specimens showed higher strain homogeneity than the one-way cold-rolled specimens. When annealed below the *Ac*_1_ temperature, two-way cold rolling accelerated recrystallization in specimen P, but not in specimen M. In the intercritically annealed specimen P, two-way cold rolling increased the average size of recrystallized ferrite grains while reducing their aspect ratio. In addition, the strength–ductility balance of the two-way cold-rolled specimen P was similar to that of the one-way cold-rolled specimen P. In contrast, in the intercritically annealed specimen M, two-way cold rolling reduced the average size and the aspect ratio of recrystallized ferrite grains. As a result, the strength–ductility balance of the two-way cold-rolled specimen M was improved by approximately 15% compared with that of the one-way cold-rolled specimen. This improvement was attributed to the formation of fine and equiaxed recrystallized ferrite grains. The present findings provide a basis for applying two-way cold rolling as a microstructure-control strategy in high-strength steels.

## 1. Introduction

Low-carbon steels are widely used in industry, especially in the automotive field. In recent years, high-strength low-carbon steels have been employed to improve the crashworthiness of suspension and structural parts [1,2]. Importantly, increases in the strength of low-carbon steel lead to a decrease in ductility, which can result in cracking during press forming. Therefore, the strength–ductility balance of low-carbon steels used in the automotive industry should be improved.

The improvement of the strength–ductility balance of low-carbon steels requires the accurate control of their microstructure. It has been reported that a homogeneous microstructure can improve the strength–ductility balance of multiphase low-carbon steels [3]. One typical multiphase low-carbon steel is dual-phase (DP) steel with a microstructure consisting of ferrite and martensite. The strength–ductility balance of DP steel can be improved through the reduction of the hardness ratio of ferrite and martensite [4,5,6], homogenization of martensite distribution [7,8,9,10], and refinement and equiaxing of ferrite grains [9,10]. For instance, Kamikawa et al. [4] employed the precipitation strengthening of ferrite to effectively improve the strength–ductility balance of vanadium-added DP steels. Furthermore, in our previous study, we revealed that fine and equiaxed recrystallized ferrite grains and the homogeneous distribution of martensite can be obtained through the annealing of steel with an initial martensite microstructure (before cold rolling and annealing) [11]. Based on these findings, in another study, we attempted to simultaneously reduce the decreasing hardness ratio of ferrite and martensite, homogenize martensite distribution, and obtain equiaxed ferrite grains in Nb-added DP steel [12]. We succeeded in dramatically improving the strength–ductility balance of the DP steel compared with conventional DP steels. Although the strength–ductility balance of DP steels has long been researched, our previous study revealed that there is still potential for improving the strength–ductility balance through relatively simple means.

Herein, we aimed to refine and obtain finer and more equiaxed recrystallized ferrite grains in DP steels. While the effects of one-way cold rolling and subsequent annealing on recrystallized ferrite grain refinement have been extensively studied, the potential of two-way cold rolling to produce a more homogeneous strain distribution—and thereby more equiaxed recrystallized ferrite grains—has not been systematically investigated in DP steels. Based on our previous findings in pure iron, two-way cold rolling can promote homogeneous nucleation of recrystallized ferrite grains during annealing [13]. Therefore, applying two-way cold rolling followed by annealing to DP steels is expected to produce finer and more equiaxed recrystallized ferrite grains, potentially leading to an improved strength–ductility balance compared with conventional DP steels. Accordingly, the purpose of the present study is to systematically clarify the effects of two-way cold rolling and subsequent annealing on microstructural evolution and tensile properties of DP steel, with a particular focus on recrystallized ferrite grain morphology and its relationship to mechanical performance.

## 2. Materials and Methods

**Figure 1:**
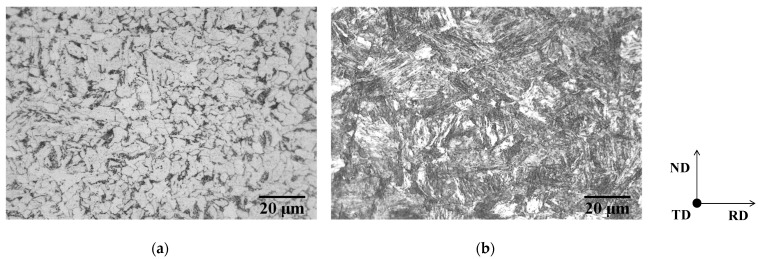
Microstructures of specimens (**a**) P and (**b**) M prior to cold rolling.

The chemical composition of the tested steels is given in Table 1. Vacuum melted ingots (approximately 100 mm in thickness) were rough-rolled to a thickness of 30 mm. The rough-rolled steels were hot-rolled at a finishing temperature of 1173 K in the austenite region to a thickness of 3.0 mm, and subsequently water cooled to 923 K (specimen P) and below 373 K (specimen M). Specimens P were furnace-cooled to room temperature (298 ± 2 K) after the water-quenching and consisted of ferrite and pearlite, and specimens M consisted of lath martensite, as shown in Figure 1. The hot-rolled specimens were first cold-rolled to a thickness of 1.80 mm in the direction perpendicular to the cold-rolling direction of the as-received sheet, and then to a thickness of 1.0 mm in the cold rolling direction of the as-received sheet (total cold-rolling reduction ratio: 67%). In some experiments, one-way cold-rolled specimens were also used, obtained through the cold rolling of hot-rolled sheets to a thickness of 1.0 mm (cold-rolling reduction ratio: 67%). The cold-rolled sheets were annealed through heating at a rate of 1 K/s to temperatures below the *Ac*_1_ temperature or to intercritical temperatures using an electric furnace, without holding at the target temperature, followed by water-quenching to room temperature. The *Ac*_1_ and *Ac*_3_ temperatures of the tested steel are approximately 943 and 1093 K, respectively. Intercritical annealing, i.e., annealing in the temperature range between *Ac*_1_ and *Ac*_3_, yielded DP steels.

The microstructures of nital-etched specimens in the rolling direction (RD)–normal direction (ND) plane were observed using optical microscopy (Shimadzu Rika Co., Ltd., Tokyo, Japan). The fraction, average size, and aspect ratio of recrystallized ferrite grains were quantitatively evaluated using the ImageJ software (version 1.54f). Additionally, Vickers hardness tests were conducted at room temperature under an applied load of 19.6 N for 10 s to evaluate the recrystallization behavior during annealing. The average values for three parallel samples are reported. The recrystallized fraction, *F_R_*, was calculated using Equation (1) [11]:

(1) $$F_{R}=\;\frac{H_{\mathrm{NR}}-H_{w}}{H_{\mathrm{NR}}-H_{R}}\times 100$$

where *H_w_* is the Vickers hardness of the entire specimen, *H_NR_* is the Vickers hardness of the as-cold-rolled specimen, and *H_R_* is the Vickers hardness of the fully recrystallized specimen. *F_R_* is commonly used as an index of the softened fraction; however, in the present study, it was used as an index of the recrystallized fraction for the sake of simplicity. A decrease in hardness during annealing in the single-phase ferrite region was mainly attributed to recrystallization. In Equation (1), *H_NR_* and *H_R_* are constant for each specimen; thus, *F_R_* increases with decreasing *H_w_*. For instance, if *H_w_* equals *H_NR_* (before the start of recrystallization), *F_R_* was calculated to be 0% using Equation (1). At the same time, if *H_w_* equals *H_R_* (immediately after the completion of recrystallization), *F_R_* was calculated to be 100% using Equation (1).

Microstructural and textural analyses in the RD–ND plane were performed on cold-rolled specimens using electron backscatter diffraction/field emission scanning electron microscopy (JSM-7100F, JEOL, Tokyo, Japan) and the orientation imaging microscopy analysis software (version 7.3, TSL solutions, Kanagawa, Japan). The step size in the backscatter diffraction measurements was 0.05 μm.

**Figure 2:**
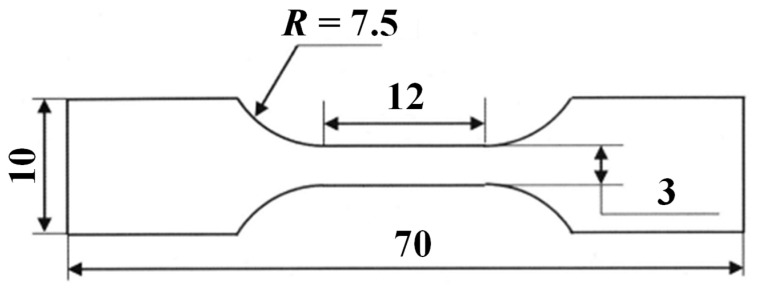
Shape of specimen for tensile testing.

The specimens were deformed at room temperature via tensile straining at a strain rate of 1.67 × 10^−3^ s^−1^. The shape of the specimen used in the tensile test is shown in Figure 2. The balance between tensile strength and total elongation was used as an index of strength–ductility balance.

## 3. Results and Discussion

### 3.1. Microstructures and Textures of Two-Way Cold-Rolled Specimens

**Figure 3:**
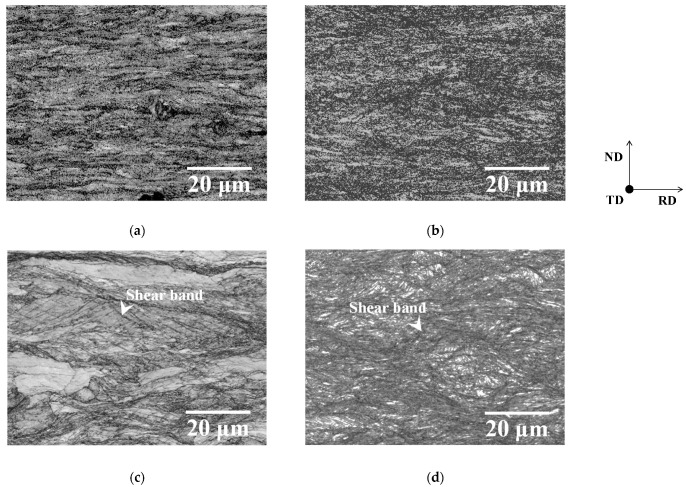
IQ maps of one-way cold-rolled specimens (**a**) P and (**b**) M and two-way cold-rolled specimens (**c**) P and (**d**) M.

**Figure 4:**
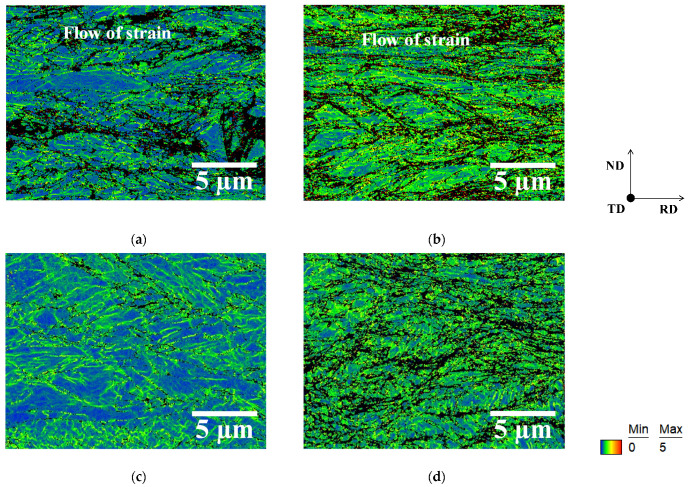
KAM maps of one-way cold-rolled specimens (**a**) P and (**b**) M and two-way cold-rolled specimens (**c**) P and (**d**) M.

Figure 3 shows image quality (IQ) maps of one-way and two-way cold-rolled specimens P and M, and Figure 4 exhibits kernel average misorientation (KAM) maps of these specimens. The regions with higher dislocation density can be recognized by a dark color, a low IQ, and a low confidence index. KAM values have been previously reported to increase with dislocation density (i.e., strain) [14]. Initial microstructure was found to have a greater effect on the KAM values of specimen M than on those of specimen P, irrespective of cold rolling direction. The IQ maps also indicate that cold rolling introduced more strain into specimen M than into specimen P. These results agree with those reported in our previous study [15]. At the same time, cold rolling direction has almost the same effects on the KAM values of the one-way and two-way cold-rolled specimens, irrespective of the initial microstructure. However, as shown in Figure 3b,d, two-way cold rolling accelerated the formation of shear bands, irrespective of the initial microstructure. It has been previously demonstrated that two-way cold rolling accelerates the formation of shear bands in pure iron [13] and Fe–Si alloys [16]. Therefore, two-way cold rolling appears to have a limited effect on dislocation density in low-carbon steel, but a pronounced effect on the formation of shear bands. In particular, as shown in Figure 3a,c, the formation of shear bands is more pronounced in the two-way rolled specimen P than in the one-way rolled specimen P. It has been previously reported that the presence of solute carbon can promote the formation of shear bands [17], meaning that shear bands are more likely to form in the one-way cold-rolled specimen M than in the one-way cold-rolled specimen P. Thus, two-way cold rolling is likely to promote the formation of shear bands in specimen P.

Next, the homogeneity of the strain introduced by cold rolling should be discussed. As shown in Figure 4, strain homogeneity in two-way cold-rolled specimens P and M is higher than that in one-way cold-rolled specimens P and M. In particular, the one-way cold-rolled specimens P and M exhibit a flow of strain in the RD (Figure 4a,b), with a notable strain heterogeneity. Here, a flow of strain and shear bands are distinct phenomena. In this study, we define a flow of strain as a band-like feature formed by the connectivity of regions (measurement points) exhibiting high KAM values. In contrast, the flow of strain in various directions, rather than only in RD, is observed in the two-way cold-rolled specimens P and M (Figure 4c,d). We have previously shown that cross cold rolling produces a rather homogeneous strain distribution in pure iron [13]. The results obtained herein agree with this previous finding.

**Figure 5:**
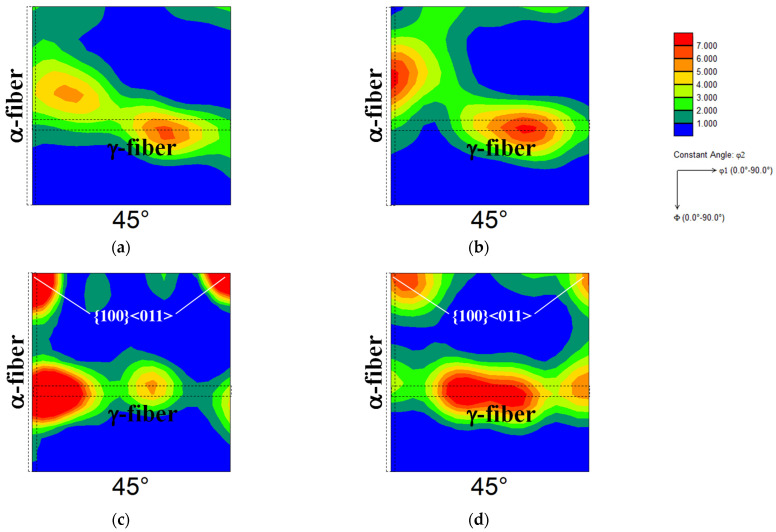
Orientation distribution function maps of one-way cold-rolled specimens (**a**) P and (**b**) M and two-way cold-rolled specimens (**c**) P and (**d**) M.

Finally, the textures of the cold-rolled specimens should be discussed. Figure 5 shows the orientation distribution function maps of one-way and two-way cold-rolled specimens P and M. In the case of one-way cold rolling, the {111}//ND fiber texture (γ-fiber) and the <110>//RD fiber texture (α-fiber) were detected in specimens P and M. This result is consistent with prior reports [13,18]. For two-way cold rolling, the formation of γ-fiber was also detected, and the development of {100}<011> orientation was observed in specimens P and M. Gobernado et al. [19] showed that cross cold rolling produces the {100}<011> orientation in interstitial-free steel. Therefore, the textures of the cold-rolled specimens obtained in this study are expected.

### 3.2. Microstructural Evolution During Annealing

**Figure 6:**
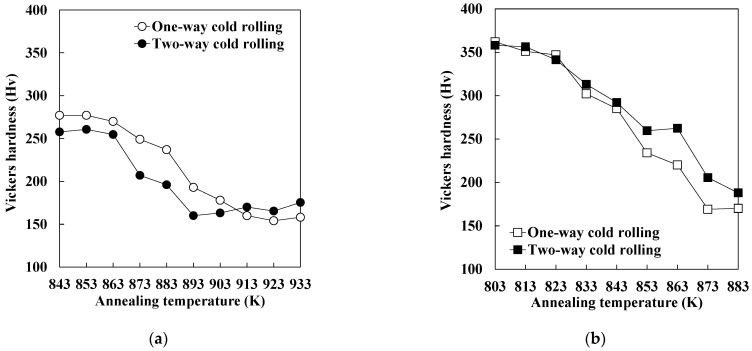
Changes in the Vickers hardness of specimens (**a**) P and (**b**) M during heating (data on one-way cold rolling were taken from a previous report [11]).

Figure 6 shows the changes in the Vickers hardness of each specimen during annealing below the *Ac*_1_ temperature. The data for one-way cold-rolled specimens shown in Figure 6 were taken from our previous study [11]. The hardness values of as-cold-rolled specimens P subjected to one-way and two-way rolling were 268 and 260 Hv, respectively. Moreover, the hardness values of as-cold-rolled specimens M subjected to one-way and two-way rolling were 479 and 490 Hv, respectively. Therefore, the amount of strain introduced by one-way and two-way cold rolling is considered to be nearly the same. This result is consistent with the data shown in Figure 3 and Figure 4. For specimen P (Figure 6a), hardness rapidly decreases when the annealing temperature exceeds 863 K, irrespective of cold rolling direction. Moreover, the hardness of one-way and two-way cold-rolled specimens stops decreasing during annealing at 923 and 893 K, respectively. In the case of two-way cold-rolled specimen P, the increase in hardness observed between 893 and 933 K is attributed to the annealing temperature approaching the *Ac*_1_ temperature, which resulted in the formation of a small amount of austenite that transformed into martensite upon cooling. At the same time, the changes in the hardness of one-way and two-way cold-rolled specimens M during annealing are similar (Figure 6b).

**Figure 7:**
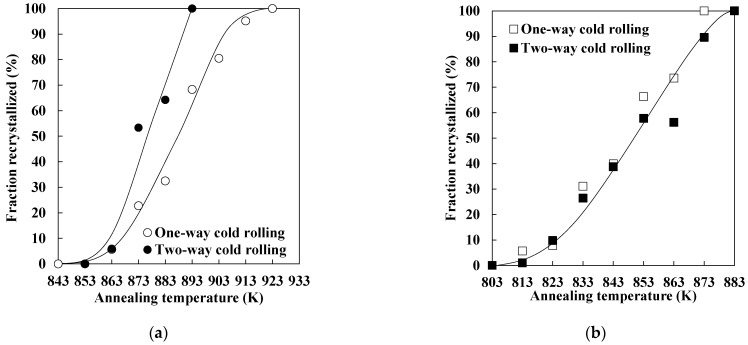
Changes in the recrystallized fraction of specimens (**a**) P and (**b**) M during heating (data on one-way cold rolling were taken from a previous report [11]).

Figure 7 shows the relationship between the recrystallized fraction calculated using Equation (1) and the annealing temperature. The data for one-way cold-rolled specimens shown in Figure 7 were taken from our previous study [11]. In the case of specimen P (Figure 7a), the recrystallization of the two-way cold-rolled specimen proceeded more rapidly than that of the one-way cold-rolled specimen. In contrast, the one-way and two-way cold-rolled specimens M show almost the same recrystallization behavior (Figure 7b). These results indicate that two-way cold rolling accelerated recrystallization during annealing in specimen P but not in specimen M. In the case of specimen P, the effect of the cold rolling direction on the amount of strain is small. However, the formation of shear bands is more pronounced in the two-way rolled specimen P than in the one-way rolled specimen P (Figure 3a,c), which may have also promoted recrystallization. We have previously demonstrated that the formation of shear bands in pure iron accelerates the nucleation of recrystallized ferrite grains during annealing [13]. For specimen M, the effect of the cold rolling direction on the amount of strain is also small. Moreover, as shown in Figure 3b,d, two-way cold rolling slightly promoted the formation of shear bands in specimen M, although the effect is weaker than that in specimen P. Therefore, the recrystallization behavior of one-way and two-way cold-rolled specimen M was likely to be similar.

**Figure 8:**
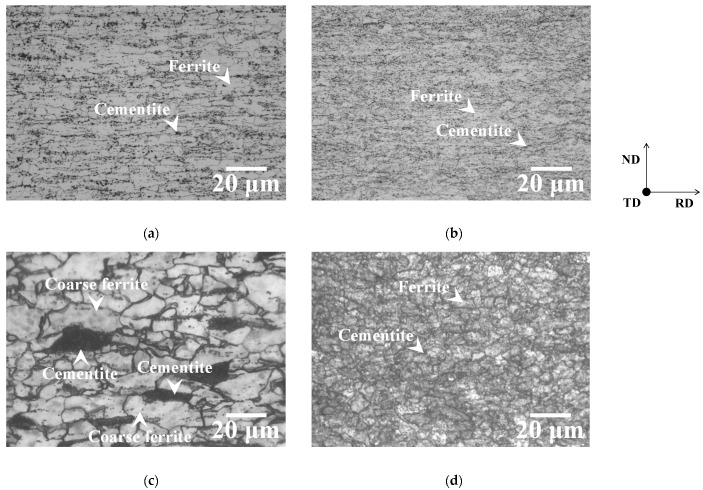
Microstructures of one-way cold-rolled specimens (**a**) P and (**b**) M and two-way cold-rolled specimens (**c**) P and (**d**) M immediately after the completion of ferrite recrystallization.

Microstructures of specimens annealed below the *Ac*_1_ temperature immediately after recrystallization completion are shown in Figure 8. The microstructures of annealed specimens are composed of ferrite and cementite particles. In the case of specimen P (Figure 8a,c), recrystallized ferrite grains in the two-way cold-rolled specimen are larger than those in the one-way cold-rolled specimen. As shown in Figure 8c, the recrystallized ferrite grains formed at ferrite–pearlite interfaces are particularly coarse. It has been previously reported that the cold rolling of ferrite–pearlite steel tends to concentrate the strain at ferrite–pearlite interfaces [20]. Moreover, as mentioned above, two-way cold rolling may have promoted the formation of shear bands. These findings suggest that two-way cold rolling may have promoted local strain concentration at the ferrite–pearlite interfaces. Therefore, two-way cold rolling can accelerate recrystallization at the ferrite–pearlite interfaces, thereby causing the coarsening of recrystallized ferrite grains. In contrast, the one-way and two-way cold-rolled specimens M exhibit little microstructural differences (Figure 8b,d).

### 3.3. Microstructures and Tensile Properties of Intercritically Annealed Specimens

**Figure 9:**
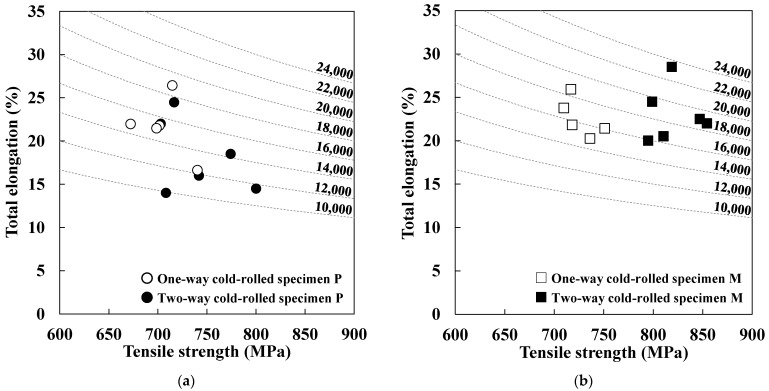
Relationship between tensile strength and total elongation in the intercritically annealed specimens (**a**) P and (**b**) M (data on one-way cold rolling were taken from a previous report [12]).

**Figure 10:**
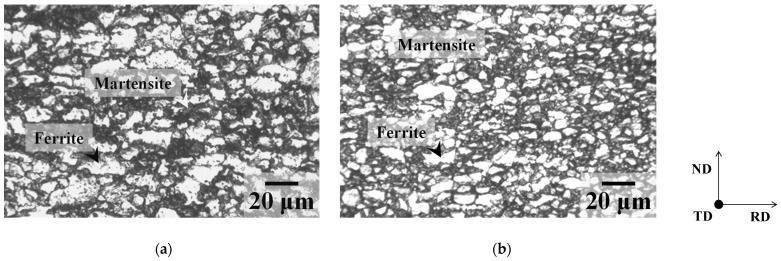
Microstructures of intercritically annealed two-way cold-rolled specimens (**a**) P and (**b**) M.

Figure 9 shows the relationship between the tensile strength and total elongation of the intercritically annealed specimens, i.e., DP steels. The values shown in Figure 9 represent the product of tensile strength and total elongation. In the case of specimens P, the strength–ductility balance in the two-way cold-rolled specimens is similar to that in the one-way cold-rolled specimens (Figure 9a). At the same time, the strength–ductility balance in the two-way cold-rolled specimens M is better than that in the one-way cold-rolled specimens M (Figure 9b). When the product of tensile strength and total elongation is used as an indicator of the strength–ductility balance, the two-way cold-rolled specimen M shows an approximately 15% improvement in this balance compared with the one-way cold-rolled specimen [12]. Specifically, the two-way cold-rolled specimens M show comparable ductility to the one-way cold-rolled specimens M, but higher strength. The microstructures of the two-way cold-rolled DP steels are shown in Figure 10. The microstructures of the one-way cold-rolled DP steels have been reported in our previous study [12]. The microstructures of DP steels are composed of ferrite and martensite, with the fraction, average size, and aspect ratio of recrystallized ferrite grains in the DP steels listed in Table 2. The values for one-way cold-rolled specimens in Table 2 were taken from our previous study [12]. We have reported that austenite is mainly formed at recrystallized ferrite grain boundaries [11,12]. In fact, the one-way cold-rolled specimen with finer recrystallized ferrite grains exhibits a slightly higher martensite fraction in specimen P. In contrast, the two-way cold-rolled specimen shows a slightly higher fraction in specimen M (Table 2). However, the volume fraction of the ferrite phase ranges approximately from 55% to 60% in all specimens. In addition, the size of martensite was almost the same in all specimens. Thus, the effect of phase fraction on the tensile properties is likely small, with the characteristics of ferrite primarily affecting the tensile properties.

We have previously revealed that fine and equiaxed ferrite grains improve the strength–ductility balance in DP steel [12]. In the case of specimen P, two-way cold rolling increased the average size of recrystallized ferrite grains while reducing their aspect ratio. The reason for the coarsening of recrystallized ferrite grains was already discussed in Section 3.2. Here, we focus on the reduction of the aspect ratio caused by two-way cold rolling. As discussed in Section 3.1, the homogeneity of the strain in two-way cold-rolled specimens P is higher than that in one-way cold-rolled specimens P. Thus, this strain homogeneity likely did not restrict the growth of recrystallized ferrite grains to the RD, leading to the formation of equiaxed grains. These results indicate that coarse recrystallized ferrite grains detrimentally affect the strength–ductility balance, whereas more equiaxed recrystallized ferrite grains enhance it, resulting in almost the same strength–ductility balance of the one-way and the two-way cold-rolled specimens.

In specimen M, two-way cold rolling reduced the average size and the aspect ratio of recrystallized ferrite grains. The refinement of recrystallized ferrite grains was attributed to the acceleration of shear-band formation by two-way cold rolling, which increased the number of nucleation sites for the recrystallization of ferrite grains. Furthermore, as in the case with specimen P, strain homogeneity induced by two-way cold rolling likely did not restrict the growth of recrystallized ferrite grains to the RD, leading to the formation of equiaxed grains. These results suggest that finer and more equiaxed recrystallized ferrite grains improve the strength–ductility balance, resulting in a better strength–ductility balance of the two-way cold-rolled specimen.

The obtained results reveal that the effect of two-way cold rolling on the strength–ductility balance depends on the initial microstructure. The more homogeneous the initial microstructure, the greater the beneficial effect of two-way cold rolling on the strength–ductility balance. Therefore, two-way cold rolling is effective in enhancing the strength–ductility balance of DP steels with a homogeneous initial microstructure.

## 4. Conclusions

Herein, we investigated the effects of two-way cold rolling and subsequent annealing on the microstructure and tensile properties of low-carbon steel with different initial microstructures and obtained the following results:

(1) Two-way cold rolling accelerated the formation of shear bands. Furthermore, two-way cold-rolled specimens exhibited higher strain homogeneity than one-way cold-rolled specimens.
(2) In the two-way cold-rolled specimens, the formation of γ-fiber was observed, along with the development of the {100}<011> orientation.
(3) Two-way cold rolling accelerated recrystallization during annealing in specimen P but not in specimen M. In the case of specimen P, two-way cold rolling increased the average size of recrystallized ferrite grains while reducing their aspect ratio. In contrast, it reduced the average size and the aspect ratio of recrystallized ferrite grains in specimen M.
(4) In the case of intercritically annealed specimen P, the strength–ductility balance in the two-way cold-rolled specimen was similar to that in the one-way cold-rolled specimen. At the same time, in the case of intercritically annealed specimen M, the two-way cold-rolled specimen showed better strength–ductility balance than the one-way cold-rolled specimen.

The present findings provide a basis for applying two-way cold rolling as a microstructure-control strategy in high-strength steels.

## Figures and Tables

**Table 1:** Chemical composition of the tested steel (mass%).

C	Si	Mn	P	S	Nb	Al	N	O
0.10	<0.003	2.03	0.010	0.0029	<0.003	0.027	0.0030	<0.001

**Table 2:** Fraction, average size, and aspect ratio of recrystallized ferrite grains in intercritically annealed specimens.

		Fraction (%)	Average Size (μm)	Aspect Ratio
One-way [12]	Specimen P	54.9	5.52	2.68
	Specimen M	57.2	3.47	1.88
Two-way	Specimen P	59.8	7.98	1.88
	Specimen M	56.9	3.08	1.66

## Data Availability

The original contributions presented in this study are included in the article. Further inquiries can be directed to the corresponding author.
